# Serological Response and Reproductive Performance in Nellore Heifers Vaccinated Against Infectious Bovine Rhinotracheitis, Bovine Viral Diarrhea, and Leptospirosis

**DOI:** 10.3390/vaccines14100849

**Published:** 2026-09-26

**Authors:** Marcos Coura Carneiro, Alice Caroline Souza e Castro, Bruna Alves Abud Ribeiro, Mirelly de Andrade Ferreira, Guardion de Sales Filho, Gabryel Henrique Cavelani Moura, Pedro Henrique Déo, Leonardo de França e Melo, Carlos Eduardo Cardoso Consentini, Amauri Alcindo Alfieri, Juliana Torres Tomazi Fritzen, Israel Barbosa Guedes, Felipe da Silva Krawczak, Marcos Bryan Heinemann, Ana Carolina Borsanelli, José Felipe Warmling Sprícigo

**Affiliations:** 1 Graduate Program in Animal Science, School of Veterinary Medicine and Animal Science, Federal University of Goiás (UFG), Goiânia 74690-900, Goiás, Brazil; mcarneiro23@discente.ufg.br (M.C.C.); leonardo.franca@ufg.br (L.d.F.e.M.); anaborsanelli@ufg.br (A.C.B.); josespricigo@ufg.br (J.F.W.S.); 2 School of Veterinary Medicine, Federal University of Minas Gerais, Belo Horizonte 31270-901, Minas Gerais, Brazil; 3 Nova Piratininga Farm, São Miguel do Araguaia 76590-000, Goiás, Brazil; 4 GlobalGen Vet Science, São Paulo 14887-360, Brazil; 5 Laboratory of Animal Virology, Department of Preventive Veterinary Medicine, Universidade Estadual de Londrina, Londrina 86057-970, Paraná, Braziljufritzen@uel.br (J.T.T.F.); 6 Laboratory of Bacterial Zoonoses, Department of Preventive Veterinary Medicine and Animal Health, School of Veterinary Medicine and Animal Science, University of São Paulo, São Paulo 05508-270, Brazilmarcosbryan@usp.br (M.B.H.)

**Keywords:** *Bos indicus*, beef cattle, reproductive pathogens, humoral immunity, serological monitoring, sanitary challenge, herd health

## Abstract

This study evaluated the serological profile, vaccine-induced antibody response, reproductive performance, and associated reproductive factors in Nellore heifers vaccinated against bovine alphaherpesvirus 1 (BoAHV-1), bovine viral diarrhea virus (BVDV), and *Leptospira* spp. in a commercial herd in Central Brazil. Heifers (*n* = 1925) were allocated to four groups: non-vaccinated control (*n* = 485), *Leptospira*-vaccinated (*n* = 478), viral-vaccinated (*n* = 484), and combined viral–leptospiral-vaccinated (*n* = 478); a 400-heifer subset was bled before vaccination and 50 and 120 days later. Antibodies were measured by virus neutralization (BoAHV-1, BVDV) and by microscopic agglutination test (*Leptospira* spp.). Data were analyzed by generalized linear mixed models. Baseline seroprevalence was 49.8% (199/400) for BVDV, 11.2% (45/400) for BoAHV-1, and 1.2% (5/400) for the *Leptospira* serovars included in the vaccine formulation. Viral-containing vaccines induced marked seroconversion against BoAHV-1 and BVDV in seronegative heifers, sustained to 120 days. The *Leptospira* spp. response was transient, peaking at 50 days. Pregnancy per artificial insemination after the first fixed-time artificial insemination (FTAI) was 51.4% (990/1925) at 30 and 48.4% (931/1925) at 60 days, with 6.0% pregnancy loss. Cumulative pregnancy over two FTAI did not differ among groups (70.0%; 1317/1881; *p* = 0.18). Corpus luteum presence *(p* = 0.0002) and inseminator *(p* = 0.01) were associated with pregnancy outcome. Direct pathogen detection was not performed, so active infection or viral reactivation could not be ruled out. Pre-breeding vaccination induced pathogen-specific humoral responses, particularly against the viral agents, but was not associated with improved pregnancy outcome under the conditions evaluated.

## 1. Introduction

Brazil is one of the world’s leading beef producers and exporters, supplying more than 2.89 million tons of beef to over 150 countries in 2024 [1]. Despite the economic importance of this sector, reproductive efficiency remains one of the principal limiting factors for productivity in beef cattle systems, particularly in tropical production conditions. Improvements in reproductive performance, including pregnancy rates and reduction in age at first calving, are essential strategies to increase productivity, accelerate genetic gains, and improve sustainability in cow-calf operations [2,3]. In this context, fixed-time artificial insemination (FTAI) programs have been widely adopted in Brazilian beef herds; however, pregnancy rates in heifers remain variable and frequently below optimal levels.

Among the factors associated with reproductive inefficiency in cattle, infectious diseases affecting the reproductive tract play a major role, particularly bovine alphaherpesvirus 1 (BoAHV-1), bovine viral diarrhea virus (BVDV), and *Leptospira* spp., the causative agents of infectious bovine rhinotracheitis, bovine viral diarrhea, and leptospirosis, respectively. These agents are endemic in Brazilian herds, affect different stages of the reproductive process, and result in substantial economic losses in beef production systems [4,5,6]. BoAHV-1 is relevant to reproductive management because, after primary infection, the virus establishes lifelong latency in sensory ganglia and may reactivate under the stressors inherent to handling, transport, and synchronization protocols, renewing viral shedding within the herd and increasing the risk of impaired conception, embryonic loss, and abortion [7]. BVDV compromises reproductive efficiency through its effects on ovarian function, fertilization, early embryonic development, and fetal infection, and through the generation of persistently infected calves, which are the main source of viral maintenance and spread within and between herds [8,9]. *Leptospira* spp. are associated with infertility, abortion, stillbirth, the birth of weak calves, and reduced reproductive efficiency, particularly in endemic regions; chronically infected cattle may sustain renal colonization and urinary shedding despite low or undetectable antibody titers [10,11]. For these reasons, the three agents are routinely included in reproductive vaccination programs in beef cattle herds.

Serological surveys conducted in Brazil demonstrated the widespread distribution of these infectious agents in cattle populations. Previous studies have reported BoAHV-1 seroprevalence ranging from approximately 50% to over 90% in beef and dairy herds, indicating extensive viral circulation within Brazilian production systems [12,13,14]. Similarly, BVDV infection is highly prevalent in Brazilian cattle, with seropositivity frequently exceeding 50% in commercial herds, reflecting the endemic nature of the disease and the persistence of infected animals within production systems [15,16]. Leptospirosis is also widely distributed in Brazilian cattle populations, with reported seroprevalence varying from 45% to over 70%, depending on the region, management system, and diagnostic criteria employed [17,18]. Together, these infections compromise reproductive performance and contribute to substantial economic losses through decreased calf production and increased replacement rates.

Vaccination is considered one of the main preventive strategies for controlling reproductive infectious diseases in cattle herds and is widely incorporated into sanitary management programs. Commercial vaccines against BoAHV-1, BVDV, and *Leptospira* spp. are commonly used in Brazilian beef production systems with the objective of reducing pathogen circulation and minimizing reproductive losses. Nevertheless, vaccine effectiveness under field conditions may vary according to management practices, vaccination schedules, herd sanitary status, and pathogen exposure pressure [19]. In addition, the interpretation of serological responses in vaccinated populations remains challenging, since antibody titers may reflect both vaccine-induced immunity and natural exposure to infectious agents.

Although vaccination against reproductive pathogens is widely recommended in cattle production systems, field studies rarely follow the same animals from baseline serology through to reproductive outcome, and evidence in *Bos indicus* heifers in their first breeding season is particularly scarce. This matters because the response to the viral, leptospiral, and combined viral–leptospiral formulations routinely used in reproductive health programs may vary with baseline serological status, pathogen exposure pressure, animal category, and herd management. We hypothesized that pre-breeding vaccination, particularly with protocols containing viral or combined viral–leptospiral antigens, would induce pathogen-specific seroconversion in initially seronegative heifers and would be associated with improved reproductive outcomes compared with non-vaccinated controls. Accordingly, the present study evaluated, in a single commercial herd over one breeding season, the apparent seroprevalence and antibody response against BoAHV-1, BVDV, and *Leptospira* spp. under four vaccination protocols, together with reproductive performance and the reproductive factors associated with pregnancy outcome.

## 2. Materials and Methods

### 2.1. Ethical Committee

All procedures were approved by the Animal Ethics Committee (CEUA) of the Federal University of Goiás (UFG), under protocol number 055/24, in accordance with national ethical guidelines for animal experimentation.

### 2.2. Study Location and Animals

The study was conducted at Fazenda Nova Piratininga, located in São Miguel do Araguaia, Goiás State, Brazil, between October 2024 and March 2025. The farm is a cow-calf operation with approximately 50,000 breeding females that operates with internal replacement, with heifers raised on the property rather than purchased. For the present study, a total of 2000 Nellore (*Bos indicus*) heifers in their first breeding season were maintained under continuous grazing on *Andropogon gayanus* pastures, with ad libitum access to water and mineral supplementation. Animals were handled only for vaccination, reproductive management, and blood sampling. Given the scale and duration of the field trial, a small proportion of heifers were progressively excluded from specific analyses whenever management, sampling, or reproductive records were incomplete; the resulting sample size was 1925 heifers.

### 2.3. Experimental Design

The study was structured into two complementary experiments. Because heifers reached breeding age at different times during the season, animals were enrolled in four separate handling events, each bringing approximately 500 heifers to the handling facility. At each event, heifers were allocated to the four experimental groups by a predefined sequential rotation as they entered, resulting in approximately 125 heifers per group per event and approximately 500 heifers per group by the end of the enrollment period.

Experiment 1 consisted of a serum epidemiological survey. At each handling event, the first 100 heifers allocated to the groups (25 per group) had blood collected, so that across the four events a 400-heifer subset (100 per group) was evaluated serologically. This experiment aimed to determine the apparent seroprevalence of antibodies against BoAHV-1, BVDV, and *Leptospira* spp., as well as to evaluate vaccine-induced serological responses over time.

Experiment 2 evaluated the reproductive performance of heifers subjected to the different vaccination protocols. At the beginning of the experiment, the mean age of the heifers was 22.7 ± 2.1 months, and the mean body weight was 277.1 ± 18.4 kg (mean ± SD).

### 2.4. Reproductive Protocol and Gynecological Assessments

The reproductive management was conducted by the farm team and comprised a cyclicity induction protocol followed by the FTAI protocol. All hormonal products were supplied by GlobalGen Vet Science (São Paulo, Brazil). On D-50, heifers were brought to the handling facility for body weight recording. On D-24, the cyclicity induction protocol was initiated with the insertion of a 500 mg intravaginal progesterone device (Repro One^®^, GlobalGen Veterinary Science, Jaboticabal, São Paulo, Brazil). On D-12, the device was removed, and 0.5 mg of estradiol cypionate (Cipion^®^, GlobalGen Vet Science, São Paulo, Brazil) was administered intramuscularly.

All heifers underwent gynecological examination on D0 of the FTAI protocol to assess ovarian cyclicity based on the presence or absence of a corpus luteum. On that same day, the animals received 2 mg of estradiol benzoate (Syncrogen^®^, GlobalGen Vet Science, São Paulo, Brazil) intramuscularly, along with the insertion of a new intravaginal progesterone device. Heifers presenting a corpus luteum on day D0 also received 0.530 mg of cloprostenol (Induscio^®^, GlobalGen Vet Science, São Paulo, Brazil) intramuscularly.

On D8, the progesterone device was removed, and heifers received 0.530 mg of cloprostenol, 0.5 mg of estradiol cypionate, and 200 IU of equine chorionic gonadotropin (ECGen^®^, GlobalGen Vet Science, São Paulo, Brazil) intramuscularly. On D10, fixed-time artificial insemination was performed, and 8.4 µg of buserelin (Maxrelin^®^, GlobalGen Vet Science, São Paulo, Brazil) was administered intramuscularly at the time of insemination. Tail paint was applied to all heifers at device removal (D8) and evaluated at FTAI (D10); heifers showing removal of the paint were considered to have expressed estrus.

Artificial inseminations were performed by two experienced technicians using conventional semen from eight bulls with proven fertility. Pregnancy diagnosis was performed at 30 days (DG30) and reconfirmed at 60 days (DG60) after the first FTAI and at DG30 only after the second FTAI. Gynecological evaluations and pregnancy diagnoses were performed by two experienced farm veterinarians using an ultrasound device equipped with a 7.5 MHz linear transrectal transducer (DP10, Mindray, Shenzhen, China).

Body weight was recorded at D-50, D0, and 30 days after the first FTAI. Animals were not fasted before weighing and were weighed individually while restrained in the chute using an integrated scale (S3, Tru-Test, Auckland, New Zealand).

### 2.5. Vaccination Protocols and Group Organization

At the beginning of the experimental trial, heifers (*n* = 2000) were assigned to one of four experimental groups according to the vaccination protocol, following the sequential rotation described in Section 2.3, with 500 heifers per group. The antigenic composition, adjuvant system, dose volume, route of administration, and schedule of each protocol are summarized in Table 1.

The first administration was performed at D-50 in all groups, by the route and at the dose volume recommended for each formulation (Table 1). Heifers in Groups 2 and 4 received a booster dose of the same vaccine at D-24, 26 days after the first dose, whereas heifers in Group 3 received a single dose, in accordance with the primary vaccination schedule of each formulation. All animals were therefore restrained and injected at both time points, so that differences among groups cannot be attributed to handling frequency or injection-related stress.

For commercial confidentiality reasons, vaccine trade names, manufacturers, and strain designations are not disclosed; the declared antigenic and adjuvant composition of each formulation is reported in full in Table 1. BoAHV-1, BVDV, and *Leptospira* spp. were the target agents of the serological evaluation described in the following section. The remaining antigens included in the Group 3 and Group 4 formulations (bovine parainfluenza virus 3, bovine respiratory syncytial virus, and *Mannheimia haemolytica*) were not evaluated for prevalence or vaccine-induced seroconversion in this study.

### 2.6. Experiment 1: Serological Survey and Epidemiological Assessment

Serological analyses were performed at two reference laboratories: virus neutralization at the Laboratory of Animal Virology, State University of Londrina (Londrina, Brazil), and the microscopic agglutination test at the Laboratory of Bacterial Zoonoses, University of São Paulo (São Paulo, Brazil). No commercial diagnostic kits were used; both assays are in-house reference techniques performed according to the WOAH Manual of Diagnostic Tests and Vaccines for Terrestrial Animals [20], using the cell cultures, viral strains, and leptospiral antigen panels routinely maintained by each laboratory.

No direct pathogen detection by PCR, virus isolation, antigen detection, bacterial culture, or shedding assessment was performed during the study. Therefore, active infection status during the experimental period was not directly determined. The results should be interpreted as antibody detection by VN and MAT, which may reflect previous exposure, natural pathogen circulation or reactivation, and/or vaccine-induced humoral responses, depending on the experimental group and sampling moment.

#### 2.6.1. Sample Size Determination and Blood Collection

Sample size was calculated from prevalence estimates using the method described by Fahim and Negida [21] assuming an expected prevalence of 50%, a 95% confidence level, and an absolute precision of 5%. Previous studies addressing the seroprevalence of BoAHV-1, BVDV, and *Leptospira* spp. in cattle were used as references. The calculation returned a minimum of 384 animals, which was rounded up to 400. A subset of 400 heifers was therefore selected from the initial study population and distributed among the four vaccination groups (100 per group) for serological evaluation.

Blood samples were collected by jugular venipuncture into 10 mL sterile evacuated tubes containing clot activator and no anticoagulant (Hosplab, São Paulo, Brazil) at three time points: D-50, immediately before the first vaccine dose (baseline); D0, the day the FTAI protocol was initiated, 50 days after the first dose; and on DG60, at the 60-day pregnancy diagnosis of the first service, 120 days after the first dose. Tubes were kept refrigerated in an insulated box with ice packs during transport and centrifuged within 6 h of collection. Samples were centrifuged at 2500 rpm (≈700× *g*) for 10 min (CE800, Centrilab, São Paulo, Brazil), and serum aliquots were stored at −20 °C until serological analysis.

#### 2.6.2. Virus Neutralization Test for BoAHV-1 and BVDV

Neutralizing antibodies against BoAHV-1 and BVDV were quantified by virus neutralization (VN) in flat-bottomed 96-well microplates. Serum samples were heat-inactivated at 56 °C for 30 min and then serially diluted two-fold in cell culture medium, for BoAHV-1 from 1:2 to 1:128 and for BVDV from 1:8 to 1:512. Titers were determined against the Los Angeles strain of BoAHV-1 and the NADL strain of BVDV, both propagated in Madin–Darby bovine kidney (MDBK) cells. Each serum dilution (50 µL) was mixed with an equal volume of viral suspension standardized to 100 TCID_50_ per well and incubated for 1 h at 37 °C in a 5% CO_2_ atmosphere. Thereafter, 50 µL of an MDBK cell suspension at 3 × 10^5^ cells/mL was added to each well, and plates were incubated at 37 °C in a 5% CO_2_ atmosphere. Results were read after 72 h under an inverted microscope, based on the presence or absence of virus-induced cytopathic effect (CPE). The neutralizing antibody titer was defined as the reciprocal of the highest serum dilution that completely neutralized viral replication, and samples with titers ≥ 1:2 for BoAHV-1 and ≥1:8 for BVDV were considered seropositive [20]. Samples were tested in a single replicate.

Each assay included a cell control (MDBK cells and medium only), a virus control (virus and cells in the absence of serum) to confirm the expected cytopathic effect, and a serum cytotoxicity control (each serum incubated with cells in the absence of virus) to verify that the serum itself did not damage the monolayer. Reference positive and negative bovine sera were included in each plate.

#### 2.6.3. Microscopic Agglutination Test for *Leptospira* spp.

Antibodies against *Leptospira* spp. were detected by the microscopic agglutination test (MAT). The panel comprised 24 live antigens representing 23 serovars from 18 serogroups, including reference strains and Brazilian isolates: Australis (serovars Australis and Bratislava), Autumnalis (Autumnalis and Butembo), Ballum (Castellonis), Bataviae (Bataviae), Canicola (Canicola), Celledoni (Whitcombi), Cynopteri (Cynopteri), Djasiman (Sentot), Grippotyphosa (Grippotyphosa), Hebdomadis (Hebdomadis), Icterohaemorrhagiae (Copenhageni and Icterohaemorrhagiae), Javanica (Javanica), Panama (Panama), Pomona (Pomona; two antigen strains), Pyrogenes (Pyrogenes), Sejroe (Guaricura, Hardjobovis, and Hardjoprajitno), Shermani (Shermani), and Tarassovi (Tarassovi).

Five serovars in the panel (Canicola, Grippotyphosa, Hardjoprajitno, Icterohaemorrhagiae, and Pomona) corresponded to those included in the leptospiral vaccine formulation and were represented by six antigens, since two antigen strains of serovar Pomona were included.

Serum samples were initially diluted 1:50 in Sörensen phosphate-buffered saline (pH 7.4) and mixed with an equal volume of antigen suspension, giving a final screening dilution of 1:100. Reactions were read under dark-field microscopy. Samples showing ≥50% agglutination at the screening dilution were considered reactive and were subsequently titrated in two-fold serial dilutions. The titer was defined as the reciprocal of the highest dilution showing at least 50% agglutination, and samples with titers ≥ 1:100 were considered seropositive [20].

### 2.7. Experiment 2: Reproductive Performance Evaluation

Reproductive performance was evaluated in heifers that completed the reproductive protocol and had complete management, insemination, and pregnancy-diagnosis records. A total of 1925 heifers were available for analysis (Group 1, *n* = 485; Group 2, *n* = 478; Group 3, *n* = 484; Group 4, *n* = 478). The following outcomes were evaluated: (i) presence of a corpus luteum at D0, as an indicator of ovarian cyclicity at the onset of the FTAI protocol; (ii) pregnancy per artificial insemination (P/AI) 30 and 60 days after the first FTAI; (iii) pregnancy loss between the two pregnancy diagnoses of the first service; (iv) P/AI 30 days after the second, resynchronized service; and (v) cumulative pregnancy.

P/AI at 30 days was defined as the proportion of heifers diagnosed as pregnant 30 days after FTAI relative to the number of inseminated heifers, and P/AI at 60 days as the proportion with pregnancy confirmed at the 60-day diagnosis relative to the same denominator. Pregnancy loss was calculated as the proportion of heifers diagnosed as pregnant at 30 days that were no longer pregnant at the 60-day diagnosis. Heifers diagnosed as non-pregnant 30 days after the first FTAI were resynchronized on the day of the non-pregnancy diagnosis, which corresponded to D0 of a second FTAI protocol. On that day, heifers received a new intravaginal progesterone device and 2 mg of estradiol benzoate intramuscularly, and the protocol then proceeded as described in Section 2.4, with the second FTAI performed 10 days later. Pregnancy was diagnosed 30 days after the second insemination; no 60-day diagnosis was performed for the second service, and pregnancy loss was therefore not estimated for it. Cumulative pregnancy was defined as the proportion of heifers pregnant either at the 60-day diagnosis of the first service or at the 30-day diagnosis of the second service, relative to the number of heifers inseminated at the first service.

The overall experimental design, including group allocation, vaccination schedule, blood sampling moments, cyclicity induction protocol, FTAI protocol, artificial insemination, resynchronization, and pregnancy diagnoses, is summarized in Figure 1.

### 2.8. Statistical Analyses

Statistical analyses were performed using SAS^®^ software (version 9.4; SAS Institute Inc., Cary, NC, USA). Descriptive data for age and body weight at the beginning of the experiment are presented as mean ± standard deviation. Continuous variables were analyzed by linear models with experimental group as a fixed effect, and model estimates are presented as least-squares means ± standard error of the mean. Binomial variables were analyzed by the PROC GLIMMIX procedure, assuming a binomial distribution and a logit link function. Apparent seroprevalences and proportions of seropositive animals are reported with 95% confidence intervals calculated by the Wilson score method.

For Experiment 1, the variables analyzed were seropositivity and seroconversion against BoAHV-1, BVDV, and *Leptospira* spp., with experimental group, sampling moment (D-50, D0, and DG60), and their interaction as fixed effects; animal was included as a repeated-measures effect to account for the three serum samples collected from each heifer.

For Experiment 2, corpus luteum presence at D0, pregnancy per artificial insemination (P/AI) at 30 and 60 days, and pregnancy loss were analyzed for the first FTAI service; only P/AI at 30 days was analyzed for the second service, since heifers resynchronized after the first service had no 60-day diagnosis and, therefore, no corresponding pregnancy-loss estimate. Cumulative pregnancy (heifers pregnant at DG60 of the first service or at DG30 of the second service) was analyzed using the same binomial model. Experimental group and inseminator were included as fixed effects, and sire as a random effect. The association of corpus luteum presence at D0 and estrus expression with P/AI at 30 days after the first FTAI was evaluated in a separate model.

Pairwise comparisons among experimental groups were performed by the Tukey–Kramer test. Differences were considered significant at *p* < 0.05, and values of 0.05 ≤ *p* < 0.10 were considered a tendency.

## 3. Results

### 3.1. Serological Profile and Vaccine-Induced Antibody Response

The baseline serological profile of the heifers is shown in Figure 2, which presents the apparent seroprevalence of antibodies against the three reproductive pathogens evaluated at D-50. Among the investigated agents, BVDV showed the highest apparent seroprevalence, with 49.8% of heifers testing positive (95% CI 44.9–54.6), followed by BoAHV-1, with 11.2% (95% CI 8.5–14.7), and the *Leptospira* serovars included in the vaccine formulation, with 1.2% (95% CI 0.5–2.9). These findings indicate distinct baseline serological profiles among the evaluated pathogens, suggesting greater previous exposure or sanitary challenge related to BVDV in the studied herd at the beginning of the experimental period.

At the first sampling time point, corresponding to D-50 of the experimental design, the apparent seroprevalence of antibodies against the vaccine *Leptospira* serovars was low in the evaluated population (Figure 2). Only five of the 400 heifers tested positive by MAT, suggesting limited detectable baseline exposure to leptospiral antigens before vaccination. Among the seropositive animals, three were positive for Pomona and two for Hardjoprajitno.

The distribution of serological responses to the vaccine *Leptospira* serovars at D0 is shown in Figure 3. This analysis compared Groups 2 and 4, both of which received vaccines containing leptospiral antigens, to identify which serovars showed detectable post-vaccination serological responses. Among the evaluated serovars, the highest seropositivity was observed for Pomona, followed by the second Pomona antigen strain (Pomona GR6) and Grippotyphosa, particularly in Group 2. Lower frequencies of seropositive animals were detected for Canicola, Icterohaemorrhagiae, and Hardjoprajitno. No significant differences *(p* > 0.05) were observed between Groups 2 and 4 for any of the evaluated serovars (Canicola, *p* = 0.18; Grippotyphosa, *p* = 0.54; Icterohaemorrhagiae, *p* = 1.00; Pomona, *p* = 0.07; Pomona GR6, *p* = 0.14; Hardjoprajitno, *p* = 0.15).

The dynamics of BoAHV-1 seropositivity throughout the experimental period are presented in Figure 4. In Panel A, which includes all evaluated heifers, the overall apparent seroprevalence at D-50 was 11.2%. At this baseline moment, seropositivity did not differ among groups (Group 1, 15.0% [15/100]; Group 2, 15.0% [15/100]; Group 3, 5.0% [5/100]; Group 4, 10.0% [10/100]; *p* = 0.20). During the experimental period, Group 1 remained at 9.2% (9/98) at D0 and 17.5% (17/97) at DG60, and Group 2 at 17.7% (17/96) at D0 and 35.8% (34/95) at DG60. In contrast, Groups 3 and 4, which received vaccines containing viral antigens, showed a marked increase in seropositivity after vaccination: Group 3 reached 97.9% (95/97) at D0 and 98.9% (91/92) at DG60, and Group 4 reached 90.9% (90/99) at D0 and 93.8% (91/97) at DG60 (*p* < 0.001 at both D0 and DG60).

In Figure 4B, the analysis was restricted to heifers that were seronegative for BoAHV-1 at D-50, allowing a more specific interpretation of post-vaccination seroconversion. Within this subgroup, Groups 3 and 4, which received vaccines containing viral antigens, exhibited a marked increase in seropositivity at D0, followed by sustained high levels of seropositivity at DG60 *(p* < 0.001 at both D0 and DG60). In contrast, Groups 1 and 2 maintained lower percentages of seropositive animals throughout the experimental period. These results support a strong vaccine-induced humoral response in initially seronegative heifers vaccinated with BoAHV-1-containing formulations, with detectable antibodies maintained until DG60.

The dynamics of BVDV seropositivity over the experimental period are shown in Figure 5. In Panel A, which includes all heifers evaluated, the overall apparent seroprevalence at D-50 was 49.8%, with no significant difference among groups (*p* = 0.38). During the experimental period, Group 1 remained at 53.1% (52/98) at D0 and 53.6% (52/97) at DG60, and Group 2 remained at 62.5% (60/96) at D0 and 61.1% (58/95) at DG60, showing only slight variations relative to baseline. In contrast, Groups 3 and 4, which received vaccines containing BVDV antigens, showed a marked increase in seropositivity after vaccination: Group 3 reached 100.0% (97/97) at D0 and 100.0% (92/92) at DG60, and Group 4 reached 94.9% (94/99) at D0 and 97.9% (95/97) at DG60 *(p* < 0.001 at both D0 and DG60).

In Figure 5B, the analysis included only heifers that were seronegative for BVDV at D-50, allowing a more specific evaluation of vaccine-induced seroconversion. In this subgroup, Groups 3 and 4 showed a strong increase in seropositivity at D0, reaching 100.0% (53/53) and 90.4% (47/52), respectively, with maintenance of high seropositivity at DG60, when Group 3 remained at 100.0% (50/50) and Group 4 reached 98.0% (49/50) *(p* < 0.001 at both D0 and DG60). In contrast, Groups 1 and 2 showed low percentages of animals becoming seropositive throughout the study: Group 1 remained at 6.2% (3/48) at both D0 and DG60, and Group 2 remained at 12.2% (5/41) at D0 and 9.8% (4/41) at DG60. These findings support a strong and sustained vaccine-induced humoral response in initially seronegative heifers vaccinated with BVDV-containing formulations.

### 3.2. Reproductive Performance After the First FTAI

Reproductive performance and baseline characteristics of heifers according to experimental group are presented in Table 2. A total of 1925 *Bos indicus* heifers, with a mean age of 22.7 ± 2.1 months and a mean body weight of 277.1 ± 18.4 kg (mean ± SD) at enrollment (D-50), were included in the reproductive analysis and allocated to Group 1 (*n* = 485), Group 2 (*n* = 478), Group 3 (*n* = 484), and Group 4 (*n* = 478).

Body weight at D-50 differed among groups (*p* = 0.01; Table 2), although the largest difference between groups was 3.5 kg, corresponding to 1.3% of the overall mean; no differences among groups were observed at D0 (*p* = 0.33). The overall proportion of heifers with a corpus luteum at D0 was 67.1% (1292/1925; 95% CI 65.0–69.2), with a tendency for a group effect *(p* = 0.07), mainly due to a higher proportion in Group 1 compared with Group 2, while Groups 3 and 4 showed intermediate values.

Regarding reproductive outcomes, the overall pregnancy per artificial insemination (P/AI) was 51.4% (990/1925; 95% CI 49.2–53.7) at 30 days and 48.4% (931/1925; 95% CI 46.1–50.6) at 60 days after FTAI. At 30 days, a tendency for a group effect was observed *(p* = 0.09), with Group 1 showing the highest P/AI and Group 3 the lowest, whereas Groups 2 and 4 showed intermediate values. At 60 days, a significant group effect was detected *(p* = 0.04), with Group 1 maintaining the highest P/AI and Group 3 showing the lowest value, while Groups 2 and 4 remained intermediate. Despite these differences in pregnancy outcomes, pregnancy loss between the two diagnoses did not differ among groups *(p* = 0.49), with an overall loss rate of 6.0% (59/990; 95% CI 4.6–7.6).

### 3.3. Factors Associated with Pregnancy per Artificial Insemination

In addition to the experimental groups, reproductive and management-related variables were evaluated for their association with pregnancy per artificial insemination at 30 days after FTAI. The presence of a corpus luteum at D0 was significantly associated with P/AI at 30 days, as heifers with a CL had higher pregnancy rates than those without a CL [54.4% (703/1292; 95% CI 51.7–57.1) vs. 45.3% (287/633; 95% CI 41.5–49.2); *p* = 0.0002]. A significant inseminator effect was also observed, with higher P/AI in heifers inseminated by Inseminator A compared with Inseminator B [54.3% (520/957; 95% CI 51.2–57.5) vs. 48.6% (470/968; 95% CI 45.4–51.7); *p* = 0.01]. Estrus expression showed a tendency to influence pregnancy outcome, with heifers detected in estrus presenting numerically higher P/AI than those without estrus expression [51.8% (970/1874; 95% CI 49.5–54.0) vs. 39.2% (20/51; 95% CI 27.0–52.9); *p* = 0.08].

Regarding sire effect, semen from eight bulls was used in the FTAI program. No significant association was observed between sire and P/AI at 30 days *(p* = 0.18), indicating that pregnancy rates did not differ statistically among the bulls used under the conditions of this study.

### 3.4. Reproductive Performance After the Second FTAI and Cumulative Pregnancy

The reproductive performance of heifers submitted to the second FTAI is presented in Table 3. A total of 935 heifers were resynchronized and included in this analysis, allocated to Group 1 (*n* = 212), Group 2 (*n* = 236), Group 3 (*n* = 249), and Group 4 (*n* = 238). The proportion of heifers with a corpus luteum at D0 was 59.7% (558/935; 95% CI 56.5–62.8) overall, with no differences among groups *(p* = 0.56). Pregnancy per artificial insemination at 30 days was 40.3% (377/935; 95% CI 37.2–43.5) overall and did not differ among groups *(p* = 0.84).

Cumulative pregnancy, defined as a positive pregnancy diagnosis at DG60 of the first FTAI or at DG30 of the second FTAI, was 70.0% (1317/1881; 95% CI 67.9–72.0) overall and did not differ among the four experimental groups (*p* = 0.18). Cumulative pregnancy rates were 73.7% (350/475; 95% CI 69.5–77.4) in Group 1, 70.2% (327/466; 95% CI 65.9–74.1) in Group 2, 68.7% (322/469; 95% CI 64.3–72.7) in Group 3, and 67.5% (318/471; 95% CI 63.2–71.6) in Group 4. Of the 2000 heifers enrolled, 119 with incomplete diagnostic information at one or both timed inseminations were excluded from this analysis.

## 4. Discussion

The baseline serological profiles differed markedly among the three pathogens. The BVDV seroprevalence was very similar to the 50.8% reported by Ribeiro-Júnior et al. [22] in zebu beef cattle from 70 herds in Tocantins, northern Brazil, whereas that study reported much higher seropositivity for BoAHV-1 and *Leptospira* spp. (90.4% and 63.2%, respectively), indicating that epidemiological pressure varies substantially with region, herd history, animal category, and management system. BVDV is among the most relevant infectious agents affecting cattle, with reproductive consequences that may be central to its economic impact [9,23]. The low BoAHV-1 seroprevalence should not be taken to reduce the epidemiological relevance of this agent, since the virus persists for life after primary infection and establishes latency in sensory ganglia, with reactivation and shedding under stressful conditions [7], a characteristic that is relevant for interpreting the slight increase in seropositivity observed over time in the groups that received no BoAHV-1 antigen.

The apparent seroprevalence of antibodies against the vaccine *Leptospira* serovars was low before vaccination and should be interpreted with caution. Leptospirosis serology depends strongly on the antigen panel, the cut-off adopted, and the timing of sampling, and the MAT, although the reference method, is serovar-specific and has recognized limitations in sensitivity, cross-reactivity, and interpretation of titers [11]. Seronegativity does not indicate absence of infection: chronically infected cattle may maintain renal colonization and shed leptospires in urine despite low or undetectable titers, particularly in maintenance infections such as those involving *Sejroe* serogroup strains, and Libonati et al. [10] showed that cattle with culture-confirmed infection frequently remained seronegative by MAT even against their own recovered isolates.

Restricting the analysis to heifers that were seronegative before vaccination minimized the influence of pre-existing antibodies and allowed a more specific interpretation of vaccine-induced seroconversion. For BoAHV-1, the marked and sustained increase in seropositivity in Groups 3 and 4, from D0 through DG60, indicates that both viral-containing protocols were immunogenic before the breeding period. This matters beyond clinical protection, since vaccination against BoAHV-1 also reduces viral circulation and shedding and may lower the risk of abortion associated with infection [7]. The increase observed in Group 2 and the fluctuation in Group 1, neither of which received BoAHV-1 antigen, should not be read as vaccine-induced seroconversion but as natural exposure, subclinical circulation, or reactivation of latent infection. This distinction matters reproductively, because BoAHV-1 has been associated with impaired conception, embryonic loss, and abortion, with effects that depend on the route of infection, viral strain, immune status, and herd-level exposure [7].

For BVDV, interpretation required more caution because the herd already showed moderate to high baseline seropositivity, which again made the seronegative subgroup the most informative. Within it, Groups 3 and 4 showed a marked increase at D0 that was sustained at DG60. Because these animals received BVDV antigens before the FTAI protocol, this pattern most likely represents a vaccine-induced response rather than field infection, and the antibody response persisted through the early post-insemination period. This is relevant because BVDV can impair reproductive efficiency through its tissue tropism, affecting follicular dynamics, fertilization, early embryonic development, and fetal infection, and may result in persistently infected calves, the main source of viral spread between herds [8,9,24,25].

The response to *Leptospira* spp. followed a different pattern: seroconversion was detected mainly at D0 in the groups receiving leptospiral antigens and had largely disappeared by DG60. The MAT was selected because it remains the reference method for leptospiral serology and is the only widely available test that discriminates among serogroups, allowing the response to be attributed to the serovars present in the vaccine formulation and the results to be compared with the epidemiological literature. Its limitation in this specific context is not diagnostic accuracy but the kinetics of the antibody class it measures: agglutinating antibodies induced by inactivated bacterins are characteristically low and short-lived, and Martínez et al. [26] showed that IgG-ELISA detected vaccine-induced antibodies more frequently than MAT after priming and boosting. The loss of MAT reactivity at DG60 therefore reflects the expected duration of the agglutinating response rather than the absence of an immune response. Protection against leptospirosis also depends on mechanisms that no serological test measures: cattle vaccinated against *Leptospira borgpetersenii* serovar Hardjo showed reduced renal and reproductive tract colonization and reduced urinary shedding after challenge despite low or transient titers [27,28], and a combined humoral and cellular response appears to correlate with vaccine efficacy in cattle [27].

After the first FTAI, the vaccination protocols did not produce a consistent reproductive benefit. The group with the highest P/AI at both 30 and 60 days was the unvaccinated control, which also showed the highest proportion of heifers with a corpus luteum at D0, whereas Group 3 showed the lowest values despite marked seroconversion against BoAHV-1 and BVDV. Because ovarian status at the onset of the protocol influences pregnancy outcome independently of vaccination, the differences among groups in P/AI are more plausibly attributed to cyclicity at D0 than to the vaccines themselves. Pregnancy loss between the two diagnoses did not differ among groups. The number of doses did not alter this picture: Groups 2 and 4 received a priming dose and a booster 26 days later, whereas Group 3 received a single dose, and reproductive performance after the first FTAI was similar among them, indicating that neither the additional antigenic coverage nor the booster conferred a detectable reproductive advantage under the conditions evaluated.

This is consistent with field studies in which reproductive vaccination did not improve fertility immediately after immunization. Morais et al. [29] found no difference in P/AI after the first FTAI in *Bos indicus* beef females subjected to two consecutive protocols and attributed the limited short-term effect to high pre-existing immunity. Ferreira et al. likewise found no difference in pregnancy rates or losses in multiparous Nellore cows vaccinated against BoAHV-1, BVDV, and leptospirosis or against leptospirosis alone. Both settings resemble the present herd, in which baseline BVDV seroprevalence was already moderate to high. A benefit has been reported by Aono et al. [30], who observed higher pregnancy rates and lower pregnancy losses in Brazilian cow–calf systems when vaccination was initiated before the FTAI program. Differences among studies therefore likely reflect animal category, initial immune status, pathogen exposure, vaccination history, and the interval between immunization and insemination [19,29,30]. The herd studied also differs from most published settings in one important respect: it is a large operation with internal replacement, in which heifers are raised on the property rather than purchased, which limits the introduction of animals from other herds. This is consistent with the serological profile observed, in which exposure was concentrated in an agent already established in the herd rather than distributed across the three pathogens evaluated.

After resynchronization, vaccination status again was not associated with pregnancy establishment. No new allocation was performed at this stage. The 935 heifers were all the females diagnosed as non-pregnant at DG30 of the first service, so the composition of each group was determined by which animals had conceived at the first FTAI rather than by any new assignment. Body weight at the start of the second protocol differed among groups, but neither the proportion of heifers with a corpus luteum nor P/AI at 30 days did. Morais et al. [29] reported the opposite pattern at the second service in *Bos indicus* females, with higher P/AI and lower pregnancy loss in vaccinated animals, which the authors attributed to a longer interval between immunization and insemination. That benefit was not reproduced here, indicating that a longer interval does not by itself guarantee higher pregnancy rates at 30 days, particularly when the outcome evaluated is pregnancy establishment rather than its maintenance at later stages.

Vaccination was also not associated with any adverse effect. Reproductive vaccination, including administration on the day of insemination, has not impaired pregnancy rates or increased pregnancy losses under field conditions [19], and the absence of differences after the second FTAI should therefore be read as a lack of detectable benefit rather than as harm. Taken together, these results reinforce the need to interpret reproductive outcomes separately from serological ones: seroconversion confirms immunogenicity, whereas pregnancy establishment under field conditions depends on a set of non-infectious factors, including ovarian status, body condition, insemination conditions, semen fertility and the uterine environment [31,32], as well as the actual level of infectious challenge during the breeding period.

When outcomes from both inseminations were combined, cumulative pregnancy did not differ among groups, indicating that the differences observed after the first FTAI did not persist once non-pregnant heifers were given a second breeding opportunity. It remains possible that a protective value of vaccination would become evident over a longer period or under a higher level of infectious challenge than that faced by this closed herd, in which vaccination against BoAHV-1 may still contribute at the herd level by reducing viral circulation and abortion risk [7].

Some limitations and strengths should be considered together. The study was conducted in a single herd during a single breeding season, with all groups managed identically, inseminated by the same two technicians with semen from the same bulls, and it included a concurrent unvaccinated control group; serological and reproductive outcomes were followed in the same animals from before the first vaccine dose. These features remove herd, season and operator as sources of confounding, which is not always possible in field evaluations of reproductive vaccines.

With approximately 480 heifers per group, the design had 80% power to detect a difference of about nine percentage points in P/AI, whereas the improvement in pregnancy attributed to BVDV vaccination in field trials has been estimated at around 5% [33]. The absence of a detected difference is therefore compatible with a true effect smaller than the one this study could resolve. The field design also limits control over environmental exposure, subclinical pathogen circulation, nutritional variation and stressors. Finally, the study was designed to characterize serological profile, antibody response and reproductive performance; assessing infection incidence, viral load, shedding, abortion attributable to a specific agent, or leptospiral renal colonization would have required direct diagnostic methods and longitudinal sampling beyond its scope. Antibody detection therefore cannot distinguish a vaccine-induced response from previous exposure, natural infection or viral reactivation for BVDV and BoAHV-1, and for *Leptospira* spp. interpretation is further constrained by the transient nature of post-vaccinal agglutinating titers.

Future studies should combine serological monitoring with direct diagnostic methods, such as PCR, antigen detection, screening for persistently infected animals, evaluation of viral shedding, and detection of leptospiral renal or urinary shedding, and should follow reproduction through to calving, including late gestational losses, calving rate, and calf viability, to clarify the relationship between vaccine-induced seroconversion, infectious challenge, and reproductive performance in Bos indicus beef herds.

## 5. Conclusions

Pre-breeding vaccination induced pathogen-specific serological responses in *Bos indicus* heifers managed under commercial beef production conditions. Virus-based vaccines elicited marked seroconversion against BoAHV-1 and BVDV in heifers that were seronegative before vaccination, with detectable antibodies persisting until 120 days. In contrast, the response against the vaccine *Leptospira* serovars was transient and detected primarily 50 days after vaccination via the microscopic agglutination test (MAT), underscoring the need for caution when interpreting leptospirosis serology following vaccination. Baseline serological profiles also indicated prior pathogen exposure within the herd, although the level of sanitary challenge varied by agent; exposure was concentrated in BVDV, consistent with a closed herd in which replacement females are raised on the property. Despite clear evidence of a vaccine-induced humoral response, vaccination protocols did not consistently improve pregnancy rates following the first or second FTAI. Likewise, cumulative pregnancy, combining the outcomes of both timed inseminations, did not differ among groups. A key limitation of this study was the absence of direct pathogen detection, which limits the ability to determine whether active infection, natural pathogen circulation, or viral reactivation influenced antibody dynamics during the study period. In practice, seroconversion should not be taken as a predictor of reproductive gain, and in closed herds with limited introduction of new agents the reproductive return of pre-breeding vaccination may be modest, although vaccination retains recognized value within herd-health programs for controlling pathogen circulation.

## Figures and Tables

**Figure 1:**
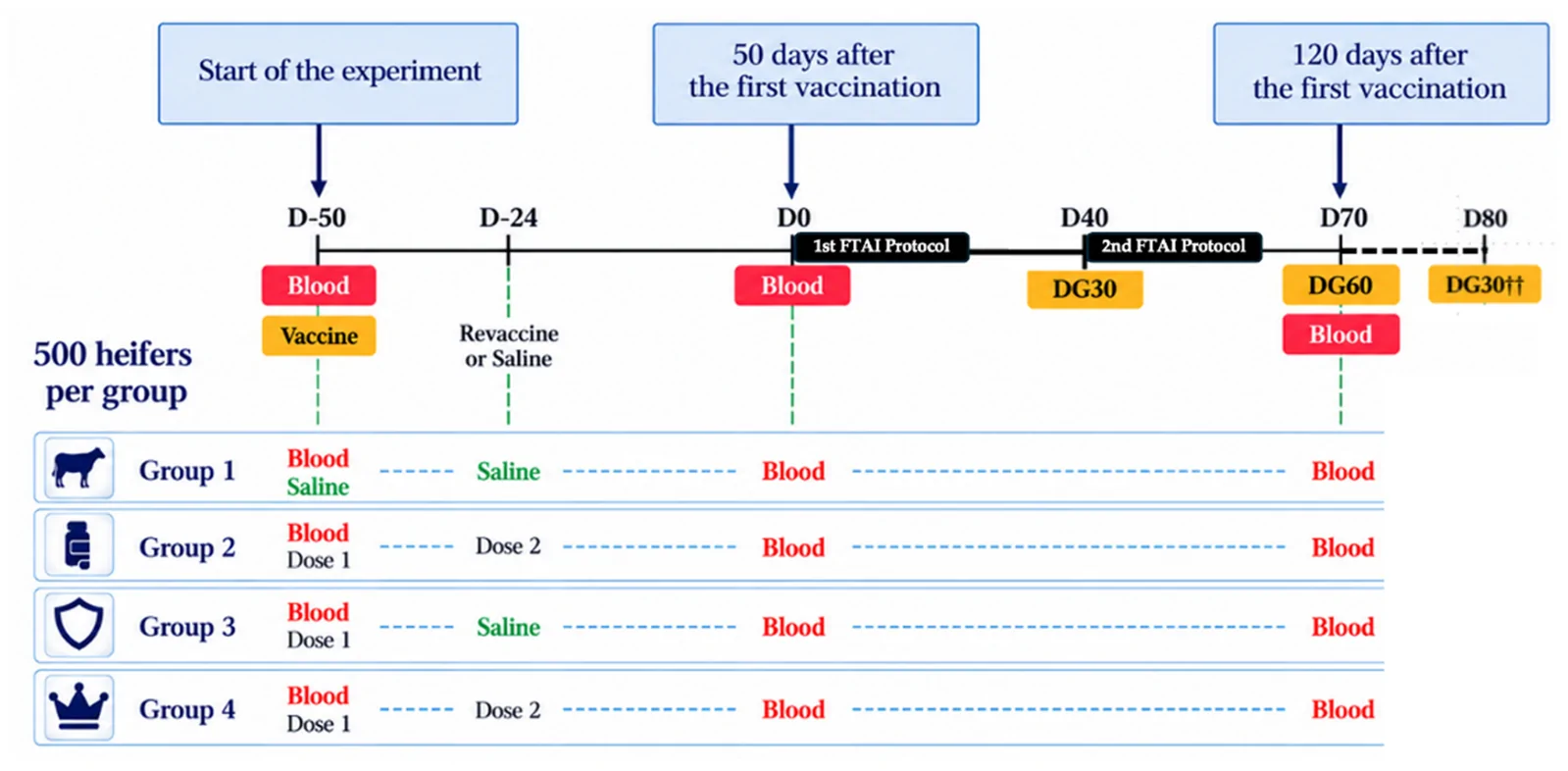
Experimental design, vaccination schedule, and blood sampling moments. The study included 500 heifers initially allocated per experimental group. Blood samples for serological analysis were collected at D-50 (before vaccination) and 50 and 120 days after the first dose, corresponding to D0 and DG60. At D-50, all groups underwent blood sampling and received the first treatment: Group 1 received saline, whereas Groups 2, 3, and 4 received the first vaccine dose. At D-24, Groups 1 and 3 received saline, while Groups 2 and 4 received the second vaccine dose. The first fixed-time artificial insemination (FTAI) protocol began at D0, with insemination at D10 and pregnancy diagnosis at D40 (DG30) and D70 (DG60). The second FTAI protocol was initiated at D40 in heifers diagnosed as non-pregnant at DG30, with insemination 10 days later and pregnancy diagnosed at D80. FTAI, fixed-time artificial insemination; DG30 and DG60, pregnancy diagnosis 30 and 60 days after insemination; ††, pregnancy diagnosis 30 days after the second insemination, performed only in heifers diagnosed as non-pregnant at DG30 of the first FTAI service.

**Figure 2:**
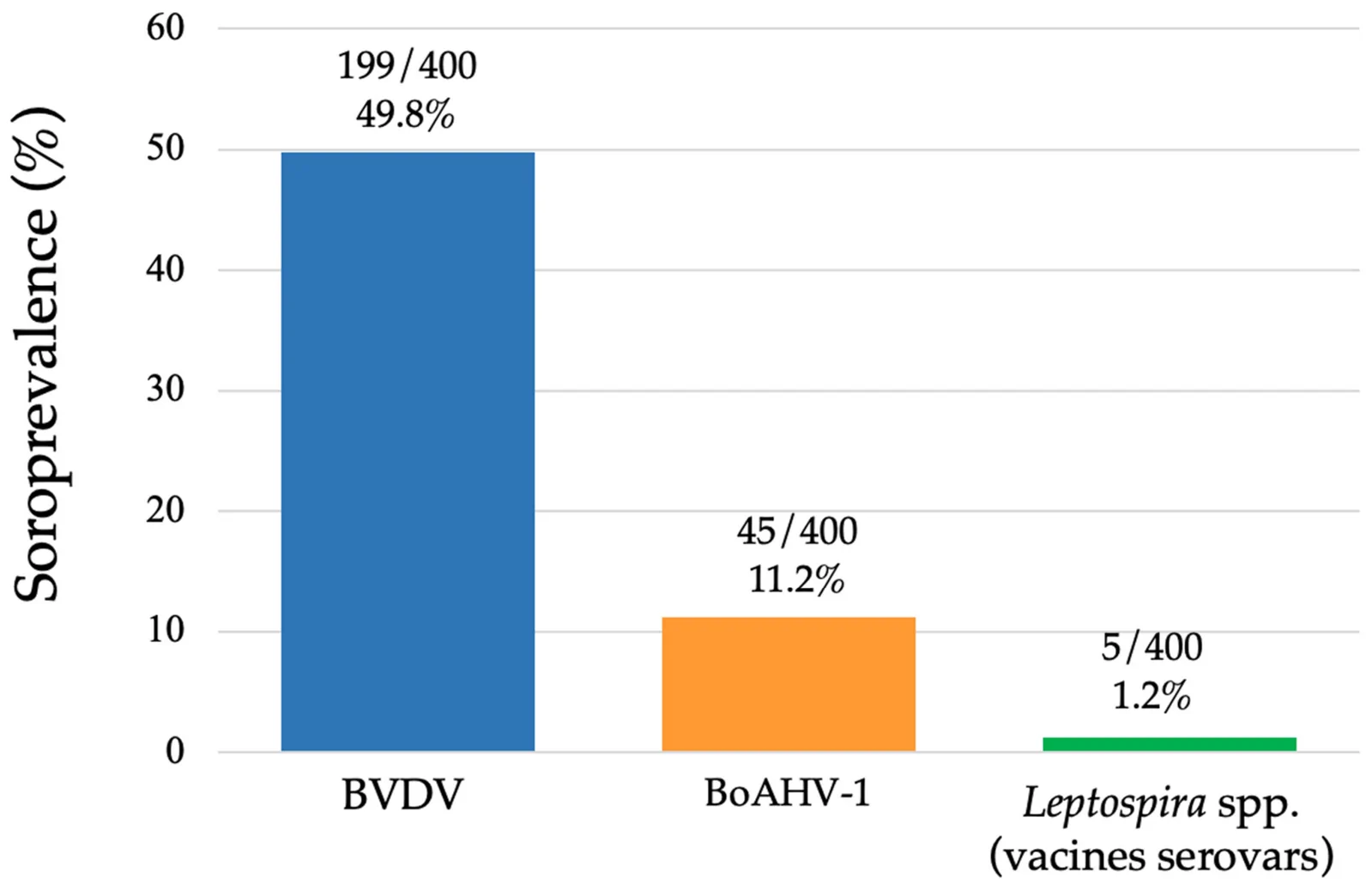
Apparent seroprevalence of antibodies against BVDV, BoAHV-1, and the *Leptospira* spp. serovars included in the vaccine formulation at D-50, before the first vaccine dose. Bars show the percentage of seropositive heifers among the 400 animals evaluated; values above the bars are n/N and the corresponding percentage. Heifers with virus neutralization titers ≥ 1:8 (BVDV) or ≥1:2 (BoAHV-1), or MAT titers ≥ 1:100 against any vaccine serovar, were considered seropositive.

**Figure 3:**
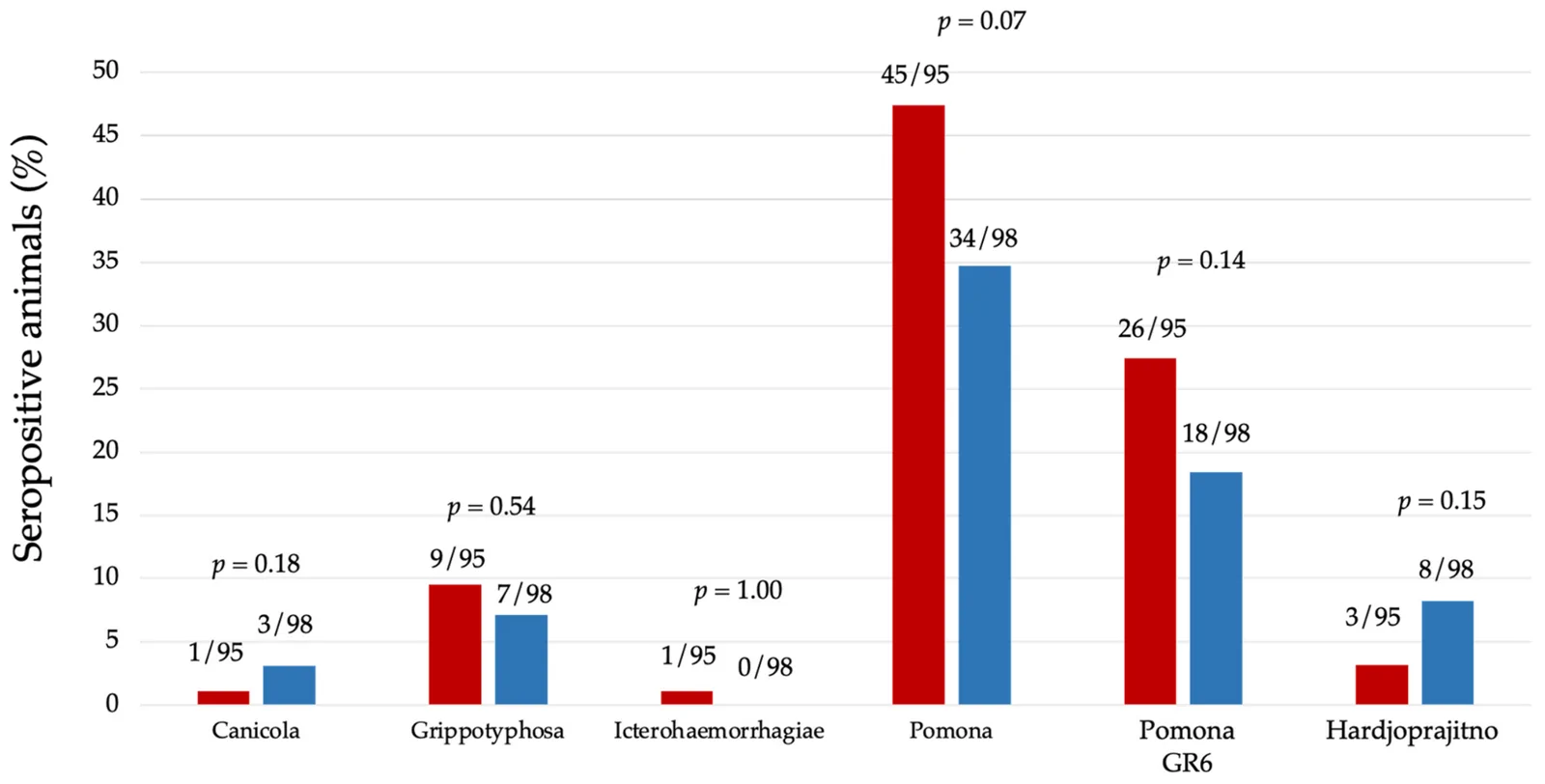
Serological response to the vaccine *Leptospira* serovars at D0 (50 days after vaccination) in the two groups that received leptospiral antigens. Bars show the percentage of seropositive heifers for each serovar; values above the bars are n/N. Red bars, Group 2 (leptospiral vaccine); blue bars, Group 4 (combined viral–leptospiral vaccine); full composition in Table 1. Horizontal lines and ***p***-values indicate the comparison between groups within each serovar. Heifers with MAT titers ≥ 1:100 were considered seropositive.

**Figure 4:**
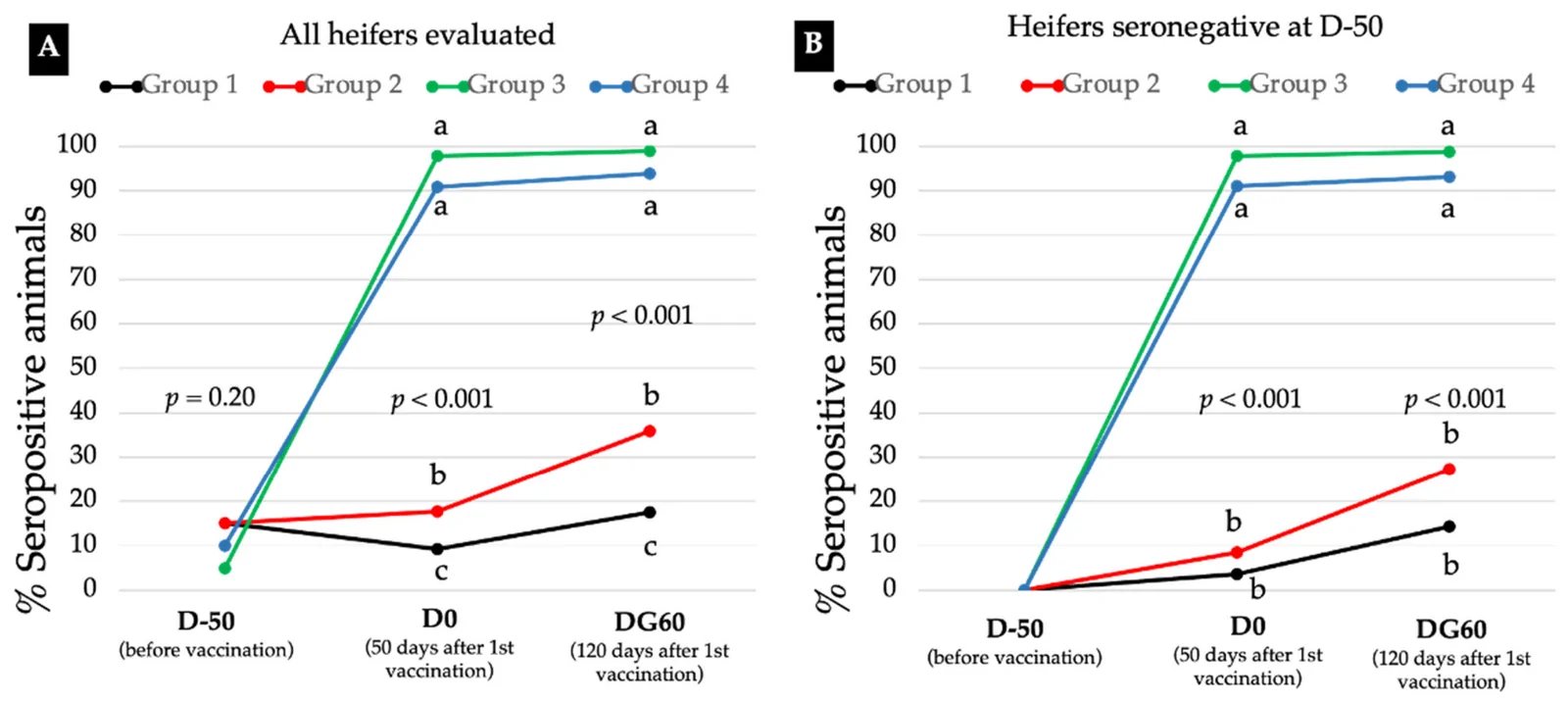
Serological dynamics of BoAHV-1 according to experimental group and sampling moment. Panel (**A**) includes all heifers evaluated (*n* = 100 per group at D-50); Panel (**B**) includes only heifers seronegative for BoAHV-1 at D-50 (Group 1, *n* = 85; Group 2, *n* = 85; Group 3, *n* = 95; Group 4, *n* = 90), allowing the evaluation of post-vaccination seroconversion. In both panels the number of heifers with serum available decreased at D0 and DG60, and the denominators for each sampling moment are given in the text. Group 1, unvaccinated control; Group 2, leptospiral vaccine; Group 3, viral vaccine; Group 4, combined viral–leptospiral vaccine (full composition in Table 1). The *p*-values refer to the comparison among groups within each sampling moment. Different lowercase letters indicate significant differences among groups within the same sampling moment (*p* < 0.05). Heifers with antibody titers ≥ 1:2 were considered seropositive.

**Figure 5:**
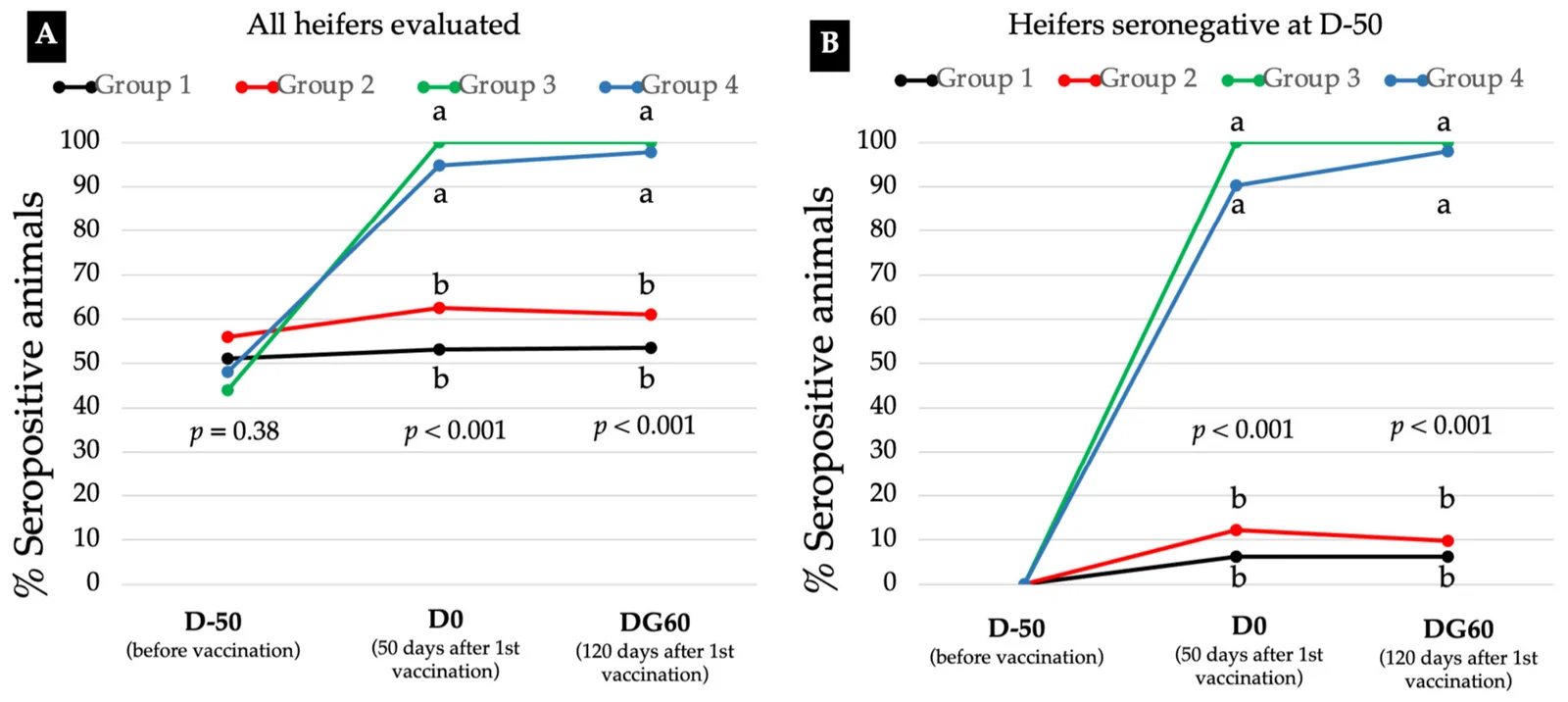
Serological dynamics of BVDV according to experimental group and sampling moment. Panel (**A**) shows the percentage of seropositive heifers considering all animals evaluated at D-50, D0, and DG60: Group 1 (*n* = 100), Group 2 (*n* = 100), Group 3 (*n* = 100), and Group 4 (*n* = 100). Panel (**B**) shows the serological response only in heifers that were seronegative for BVDV at D-50, allowing the evaluation of post-vaccination seroconversion: Group 1 (*n* = 49), Group 2 (*n* = 44), Group 3 (*n* = 56), and Group 4 (*n* = 52). Group sizes refer to D-50; denominators at D0 and DG60 vary slightly owing to unavailable samples and are reported in the text. Group 1 received saline solution; Group 2 received a vaccine containing only inactivated Leptospira spp. serovars; Group 3 received a multivalent vaccine with BoAHV-1 and BVDV among its target agents; and Group 4 received a combined vaccine containing viral antigens: BoAHV-1, PI3, BRSV, and BVDV, plus inactivated *Leptospira* spp. serovars. Different letters within the same sampling moment indicate significant differences among groups (*p* < 0.05). Animals with antibody titers ≥ 1:8 were considered seropositive.

**Table 1:** Composition, adjuvant, dose, route, and schedule of the vaccination protocols evaluated.

Group	Antigenic Composition	Adjuvant	Dose and Route	Schedule
1	Sterile saline solution (non-vaccinated control)	Not applicable	2 mL, SC	D-50 and D-24
2	Chemically inactivated *Leptospira* bacterin: serovars Canicola, Grippotyphosa, Hardjo, Icterohaemorrhagiae, and Pomona	Not declared on the product label	2 mL, IM	D-50 and D-24 (two doses)
3	Modified-live viral vaccine: BoAHV-1, BVDV-1, BVDV-2, bovine parainfluenza virus 3, and bovine respiratory syncytial virus, combined with a whole-culture preparation of *Mannheimia haemolytica*	Adjuvanted sterile diluent used to rehydrate the lyophilized fraction	2 mL, SC	D-50 (single dose); sterile saline at D-24
4	Chemically altered, temperature-sensitive BoAHV-1 and bovine parainfluenza virus 3; modified-live bovine respiratory syncytial virus; inactivated cytopathic and noncytopathic BVDV-1 and BVDV-2; chemically inactivated *Leptospira* serovars Canicola, Grippotyphosa, Hardjo, Icterohaemorrhagiae, and Pomona	Oil-in-water adjuvant system	5 mL, SC	D-50 and D-24 (two doses)

IM = intramuscular; SC = subcutaneous.

**Table 2:** Baseline characteristics and reproductive performance after the first FTAI of *Bos indicus* heifers according to experimental group.

Variable	Overall	Group 1	Group 2	Group 3	Group 4	*p*-Value
Number of animals	1925	485	478	484	478	
Age at D-50, months (means ± SEM)	22.7 ± 0.1	22.7 ± 0.1	22.7 ± 0.1	22.6 ± 0.1	22.8 ± 0.1	0.79
Body weight at D-50, kg (means ± SEM)	277.1 ± 0.4	278.8 ± 0.8 ^a^	276.5 ± 0.8 ^ab^	275.3 ± 0.8 ^b^	278.0 ± 0.9 ^ab^	0.01
Body weight at D0, kg (means ± SEM)	306.3 ± 0.5	307.1 ± 1.0	305.3 ± 1.0	305.6 ± 0.9	307.4 ± 1.0	0.33
CL at D0, % (n/N)	67.1	71.8 (348/485) ^A^	63.8 (305/478) ^B^	66.5 (322/484) ^AB^	66.3 (317/478) ^AB^	0.07
P/AI at 30 d, % (n/N)	51.4	56.3 (273/485) ^A^	50.6 (242/478) ^AB^	48.6 (235/484) ^B^	50.2 (240/478) ^AB^	0.09
P/AI at 60 d, % (n/N)	48.4	53.8 (261/485) ^a^	47.5 (227/478) ^ab^	44.8 (217/484) ^b^	47.3 (226/478) ^ab^	0.04
Pregnancy loss, % (n/N)	6.0	4.4 (12/273)	6.2 (15/242)	7.7 (18/235)	5.8 (14/240)	0.49

Group 1, unvaccinated control; Group 2, leptospiral vaccine; Group 3, viral vaccine; Group 4, combined viral–leptospiral vaccine (full composition in Table 1). Data are presented as mean ± standard error of the mean (SEM) or as a percentage followed by n/N. D-50, day of the first vaccine dose; D0, first day of the FTAI protocol. Pregnancy loss was calculated relative to the heifers diagnosed as pregnant at 30 days. CL, corpus luteum; P/AI, pregnancy per artificial insemination; FTAI, fixed-time artificial insemination. Within a row, different lowercase letters indicate significant differences among groups (*p* < 0.05) and different uppercase letters indicate tendencies (0.05 ≤ *p* < 0.10).

**Table 3:** Reproductive outcomes of the second, resynchronized FTAI service in *Bos indicus* heifers, according to experimental group.

Variable	Overall	Group 1	Group 2	Group 3	Group 4	*p*-Value
Number of animals	935	212	236	249	238	-
Body weight at D0, kg (means ± SEM)	329.5 ± 0.7	337.4 ± 1.4 ^a^	330.6 ± 1.4 ^b^	324.3 ± 1.3 ^c^	326.8 ± 1.5 ^bc^	<0.001
CL at D0, % (n/N)	59.7	63.7 (135/212)	57.2 (135/236)	59.0 (147/249)	59.2 (141/238)	0.56
P/AI at 30 d, % (n/N)	40.3	41.0 (87/212)	41.1 (97/236)	41.4 (103/249)	37.8 (90/238)	0.84

Group 1, unvaccinated control; Group 2, leptospiral vaccine; Group 3, viral vaccine; Group 4, combined viral–leptospiral vaccine (full composition in Table 1). Only heifers diagnosed as non-pregnant 30 days after the first FTAI were included. D0, first day of the second FTAI protocol, which began on the day of that diagnosis, approximately 40 days after D0 of the first protocol. Data are presented as mean ± standard error of the mean (SEM) or as a percentage followed by n/N. CL, corpus luteum; P/AI, pregnancy per artificial insemination; FTAI, fixed-time artificial insemination. Within a row, different lowercase letters indicate significant differences among groups (*p* < 0.05).

## Data Availability

The data presented in this study are available on request from the corresponding author. The data are not publicly available due to ethical and privacy restrictions related to the use of animals from a commercial herd.

## Abbreviations

The following abbreviations are used in this manuscript:

BoAHV-1	bovine alphaherpesvirus 1
BRSV	bovine respiratory syncytial virus
BVDV	bovine viral diarrhea virus
CEUA	Animal Ethics Committee (Comitê de Ética no Uso de Animais)
CI	confidence interval
CL	corpus luteum
DG30	pregnancy diagnosis 30 days after FTAI
DG60	pregnancy diagnosis 60 days after FTAI
ELISA	enzyme-linked immunosorbent assay
FTAI	fixed-time artificial insemination
GnRH	gonadotropin-releasing hormone
IBR	infectious bovine rhinotracheitis
IM	intramuscular
MAT	microscopic agglutination test
MDBK	Madin–Darby bovine kidney
P/AI	pregnancy per artificial insemination
PCR	polymerase chain reaction
PI3	bovine parainfluenza virus 3
SC	subcutaneous
SD	standard deviation
SEM	standard error of the mean
TCID_50_	50% tissue culture infectious dose
UEL	State University of Londrina
UFG	Federal University of Goiás
USP	University of São Paulo
VN	virus neutralization
WOAH	World Organisation for Animal Health
